# Cysteine-encoded chirality evolution in plasmonic rhombic dodecahedral gold nanoparticles

**DOI:** 10.1038/s41467-019-14117-x

**Published:** 2020-01-14

**Authors:** Hye-Eun Lee, Ryeong Myeong Kim, Hyo-Yong Ahn, Yoon Young Lee, Gi Hyun Byun, Sang Won Im, Jungho Mun, Junsuk Rho, Ki Tae Nam

**Affiliations:** 1 Department of Materials Science and Engineering, Seoul National University, Seoul, 08826 Korea; 2 Department of Chemical Engineering, Pohang University of Science and Technology (POSTECH), Pohang, 37673 Korea; 3 Department of Mechanical Engineering, Pohang University of Science and Technology (POSTECH), Pohang, 37673 Korea

**Keywords:** Nanoparticles, Synthesis and processing

## Abstract

Chiral plasmonic nanostructures have opened up unprecedented opportunities in optical applications. We present chirality evolution in nanoparticles focusing on the crystallographic aspects and elucidate key parameters for chiral structure formation. From a detailed understanding of chirality formation, we achieved a morphology (432 Helicoid IV) of three-dimensionally controlled chiral plasmonic nanoparticles based on the rhombic dodecahedral shape. The role of the synthesis parameters, seed, cysteine, cetyltrimethylammonium bromide and ascorbic acid on chiral formation are studied, and based on this understanding, the systematic control of the chiral structure is presented. The relation between the modulated chiral structure factors and optical response is further elucidated by electromagnetic simulation. Importantly, a new optical response is achieved by assembling chiral nanoparticles into a film. This comprehensive study of chiral nanoparticles will provide valuable insight for the further development of diverse chiral plasmonic nanostructures with fascinating properties.

## Abstract (web summary)

Chirality of amino acids can be transferred to the high index plane of gold nanoparticles, resulting in chiral plasmonic nanoparticles called the 432 Helicoid series. Here, the authors present a 432 Helicoid IV and describe a systematic approach to modulate the chirality of single particles by controlling the kinetics.

## Introduction

Understanding chiral nanostructures is of central importance in a broad range of scientific fields, including chiral recognition and separation of molecules in reactions^1^, handedness-dependent optical responses^2^, and mechanical function in chiral structures^3^. Recently, chiral structures combined with plasmonic materials have gained special attention owing to their unprecedented optical properties^4–11^. The unique characteristics of plasmonic materials that can maximize the light–matter interaction have opened up possibilities to utilize polarized light by rational design of plasmonic nanostructures^12–16^. Based on the deliberately designed structure, exciting light-manipulating abilities such as polarization modulation^17,18^, phase control^15,16^, and a negative refractive index^19^ have been achieved. To exploit the unique optical properties of chiral plasmonic nanostructures, a variety of fabrication strategies have been developed. Top–down nanofabrication approaches, such as electron-beam lithography and focused ion beam milling, offer a way to create well-defined structures with nanometer-sized dimensions^14^. Utilizing biomolecules such as DNA^20–26^, peptide^27–30^, and protein^31^ as a template for nanoparticle assembly enables the construction of asymmetric nanostructures based on the defined structure of a biomolecule. Recently, there have been multiple increasing studies on synthesizing the chiral morphology based on the interaction between chiral molecules and inorganic materials^6,8,32–37^. However, despite numerous efforts, controlling the chiral structure at the nanometer scale remains a nontrivial challenge. In particular, fabricating asymmetric nanostructures based on solution-phase synthesis and producing isotropic three-dimensional chiral structures are still difficult to achieve.

Previously, we developed a strategy to make three-dimensional chiral nanoparticles using amino acid and peptides as shape-directing additives^38^. The key requirements for chiral nanoparticle synthesis are (1) generation of a high-Miller-index surface and (2) enantioselective interaction between the molecules and surfaces. To synthesize high-Miller-index nanoparticles, we devised a seed-mediated two-step growth method. In the first growth step, 50-nm-sized low-index nanoparticles were prepared by growing spherical particles. These predefined low-index nanoparticles were further grown in a second growth solution, resulting in 150-nm-sized nanoparticles with the {321} high-index facets exposed. During the second growth step, where a high-index surface is generated, amino acids or peptides were integrated and made enantioselective interactions with the high-index surface, which has innate chirality originating from the atomic arrangement. The chiral selective interaction of the molecule and gold surface leads to asymmetric growth toward a specific direction, resulting in a distinctive chirality in the nanoparticles. By utilizing two different molecules, cysteine (Cys) and glutathione, we achieved two chiral nanoparticles, named 432 helicoids I and II, respectively. Furthermore, the change of the low-index nanoparticles from cube to octahedron led to another type of chiral-shaped nanoparticle, 432 helicoid III, which showed the highest g-factor of 0.2 in the visible range among the reported nanoparticles based on bottom-up synthesis methods.

Here, we provide a comprehensive understanding of chiral formation in this synthesis strategy. In particular, we show various synthesis factors that can determine the chirality evolution in the nanoparticles and demonstrate systematic modulation of the chiral structure using chemical parameters. From the change of the concentration and injection time of seed nanoparticles, a different intermediate shape is fabricated, and different starting points of chiral evolution are provided. This crystallographically and geometrically different basal structure allows us to fabricate a three-dimensional chiral plasmonic nanostructure based on a rhombic dodecahedron shape. Cys regulates not only enantioselectivity but also the degree of chiral deformation and kinetics of chiral development. Cetyltrimethylammonium bromide (CTAB) and ascorbic acid (AA) affect the vertical and lateral growth of the chiral edge. These factors cooperatively act on the development of the chiral structure, making a 432 helicoid structure with strong optical activity. For a quantitative understanding of the chiral structure on the optical response, several structural parameters are studied based on numerical simulation, and the degree of twist, protrusion, and width of the edge are found to be key parameters for strong optical activity. Furthermore, a new chiroptical response is created by assembling the particles on the substrate, based on the merits of the chirality developed in single nanoparticles and the high-density packing of nanoparticles. In addition, by making helicoid nanoparticles as a film type, we suggest the possibility that 432 helicoid particles can be directly applied to conventional two-dimensional platforms or further utilized to fabricate a metamaterial by combining various existing nanotechnologies.

## Results

### Synthesis of helicoid nanoparticles

In the typical synthesis of chiral plasmonic nanoparticles, a stepwise growth of the seed, named the two-step growth method, was used. First, 50-nm-sized low-Miller-index nanoparticles were prepared by growing 2 nm spherical particles in the growth solution containing the gold precursor, CTAB, and AA (first growth step). As CTAB inhibits the {100} surface while AA promotes growth along the <111> direction, tuning the ratio between CTAB and AA in the growth solution allows us to control the index of the nanoparticles^39^. By taking advantage of the CTAB and AA system, we synthesized well-defined 50-nm-sized cuboctahedron particles (Supplementary Fig. 1) and utilized them for further growth. In the second growth step, 50-nm-sized cuboctahedron seeds were grown in the presence of Cys molecules. The reaction was started by adding the seeds into the growth solution consisting of the gold precursor, CTAB, AA, and Cys and was continued for 1 h (see Methods for experimental details). Seed nanoparticles with a predefined shape evolved into nanoparticles with different indices as a result of the attachment of gold atoms from the growth solution. At the same time, during the transformation of seeds, the Cys molecules continuously attached to the gold surface and modified the growth direction via strong interaction, resulting in Cys-induced chiral nanoparticles.

**Fig. 1:**
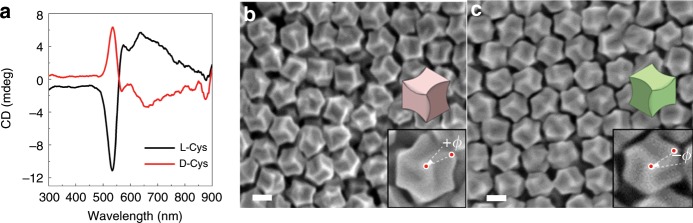
Opposite handedness of chiral nanoparticles depending on the cysteine enantiomer. **a** Circular dichroism (CD) spectra of nanoparticles synthesized using L-cysteine (L-Cys, black) and D-cysteine (D-Cys, red). SEM images of resultant chiral nanoparticles obtained using L-Cys (**b**) and D-Cys (**c**). Schematic models and magnified SEM images are shown in the inset. Auxiliary lines indicating oppositely bent edges at an angle of +*ϕ* for L-Cys and −*ϕ* for D-Cys are illustrated in the SEM images. Scale bar, 100 nm.

The circular dichroism (CD) spectra and scanning electron microscopy (SEM) images in Fig. 1 show the chiral formation in the synthesized gold nanoparticles. The handedness of the Cys determined the optical response and direction of the twists of the nanoparticles. When L-cysteine (L-Cys) was used as an additive, the resultant nanoparticles absorbed right circularly polarized light (CPL) at 534 nm and left CPL at 638 nm. On the other hand, the nanoparticles synthesized in the presence of D-cysteine (D-Cys) molecules showed an exactly inverted CD response with the same peak position, while the extinction spectrum was the same (Supplementary Fig. 2). The outline of the nanoparticles in both cases was similar to that of a rhombic dodecahedron (RD) with a size of 150 nm. The RD is composed of 12 rhombic faces and 24 edges, and it has a characteristic symmetric axis depending on the viewing direction (e.g., threefold rotational rhombuses along the [111] direction). Interestingly, a notable aspect of this structure is that the edges of the nanoparticles, which were originally straight in the RD, are twisted in opposite directions depending on the L-Cys and D-Cys. In the case of L-Cys, a 60-nm-sized edge, which connects the center to the vertex of the hexagon, was bent by an angle of +*ϕ*, as shown in the inset of Fig. 1b (yield: 94.76%, Supplementary Fig. 2). For the particle synthesized in the presence of D-Cys, the edge with the same size was bent in the opposite direction at an angle of –*ϕ*, thus imparting chirality to the resultant nanoparticles with a discernible CD signal. When the 1:1 ratio of L-Cys:D-Cys was used for synthesis, only achiral nanoparticles with no observable CD signal were achieved (Supplementary Fig. 2). These results prove that the enantiomorphic twist originated from the Cys chirality. In addition, a series of control experiments further demonstrated that the chiral structure of the nanoparticles results in the generation of CD signals (Supplementary Fig. 3 and Supplementary Discussion 1). As the synthesized chiral nanoparticle was constructed on an RD base shape, which is different from the previous 432 helicoids I, II, and III (Supplementary Fig. 4), we refer to the nanoparticle as 432 helicoid IV, a new class of helicoids.

### Chiral evolution of 432 helicoid IV

**Fig. 2:**
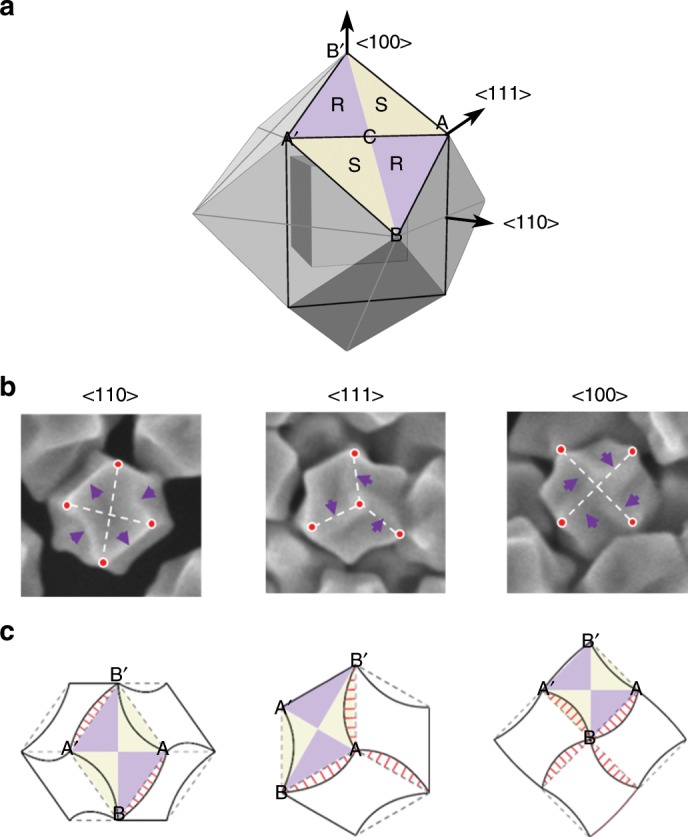
Mechanism of chiral evolution in 432 helicoid IV. **a** Overlay of a cube and rhombic dodecahedron (RD) three-dimensional model aligned in the same direction. Crystallographic <110>, <111>, and <100> directions are also shown. Representative rhombus face ABA′B′ is highlighted with a label. Prime (′) refers to the symmetrical point of the corresponding vertex. In the rhombus, a pair of R and S chiral conformations of a high-index gold surface are illustrated in purple (R region) and yellow (S region). **b** SEM images of the helicoid oriented along the <110>, <111>, and <100> axes. For clear visualization of the distorted edge, purple arrows and auxiliary white dashed lines that connect the vertices (red dots) are shown. **c** Two-dimensional schematic model of the RD (dashed gray line) and 432 helicoid IV (solid black line) viewed along three different directions. The same rhombus highlighted in panel **a** is drawn in all the schematics. The area crossing over the RD outline, the newly expanded R region, is marked with red solid lines.

To understand the chiral development of 432 helicoid IV, time-dependent evolution of nanoparticles was observed by SEM and CD measurements at 5-min intervals (Supplementary Fig. 5). After 10 min of growth, the cuboctahedron seeds transformed into the RD. During transformation of the seeds from a flat {100} facet (cube) to a {110} facet (RD), diverse combinations of these two low-index facets, namely, high-Miller-index facets, were generated (Supplementary Fig. 6). Since the Miller index of nanoparticles can be directly related to the geometrical parameters of the projection^40^, the possible Miller indices of the exposed planes were estimated from the relative angles of the facets and are listed in Supplementary Fig. 6. The generation of a high-index surface is essential for chiral evolution in nanoparticles, as high-index {hkl} planes possess innate chirality depending on the atomic arrangement. With a kink atom as a chiral center, the clockwise rotation of low-index microfacets (111), (100), and (110) around the kink generates an R-chiral conformation, while anticlockwise rotation provides an opposite chirality (S-chiral conformation)^41–43^. The formation of high-index nanoparticles leads to R- and S-chiral conformations on the surface of gold with equal distribution. The pairs of chiral R and S {hkl} facets are displayed in the RD model, wherein purple indicates R and yellow indicates S (Fig. 2a).

A discernable twist started to appear from 15 min, and as the growth proceeded, the size of the twist of the edge became larger with increasing RD size. Due to the incremental distortion, the CD intensity also gradually increased, depending on the growth time (Supplementary Fig. 5). Figure 2b and c shows the SEM images of the final morphology and the corresponding models. To clearly distinguish the distortion of the edges, red dots, dashed white lines, and arrows are delineated on the SEM images. With careful observation, diverse asymmetrical structures were identified in all three different directions of the particles. A schematic model of the RD (gray dashed line), which appeared at the early growth stage, and the chiral deformation of the particles (black solid line) aligned with the same orientation were analyzed (Fig. 2c). In the RD model, the representative rhombus ABA′B′ and the same R- and S-chiral conformations that are shown in Fig. 2a are indicated. The particle shows unique orthogonal projections of the RD and elongated hexagonal, hexagonal, and square contours corresponding to the <110>, <111>, and <100> symmetrical axes, respectively. In the <110> alignment, deformation was observed for the four outer boundaries of rhombus ABA′B′. The AB and A′B′ boundaries bent outward, while AB′ and A′B moved inward, which led to anisotropic distortion of the rhombus. This change was operated on 12 congruent rhombuses, and generated characteristic concave and convex outlines in 432 helicoid IV with twofold, threefold, and fourfold rotation axes along the <110>, <111>, and <100> directions, respectively.

Based on the detailed structural analysis in Fig. 2, the mechanism of chiral formation was elucidated. Taking into account the R and S regions together, the characteristic deformation of AB and A′B′ boundaries can be regarded as the result of expansion of the R region with simultaneous contraction of the S region. As the growth proceeds, the R region, which was originally of the same proportion as that of the S region at an early stage, expanded toward the S region, creating a chiral deformation in the nanoparticles. From these observations, we assume that the asymmetric changes in the R and S regions originated from the enantioselective interaction of the Cys molecule on the chiral gold surfaces.

Several previous reports have shown the chiral recognition between an enantiomer and metal surfaces^44–49^. Gellman’s group experimentally demonstrated an enantiospecific interaction by showing different desorption energies between a chiral molecule and the surfaces of Cu(643)^R^ and Cu(643)^S^^44^. In addition, from X-ray photoelectron spectroscopy analysis and density functional theory calculation, it was found that D-Cys binds more strongly (140 meV) than L-Cys on Au (17 11 9)^S^ and that the discriminative adsorption of the functional group of Cys is the origin of the enantioselectivity^45,46^. In addition, scanning tunneling microscope analyses directly showed the opposite adsorption geometries of chiral molecules, depending on the atomic arrangement of metal surfaces^47–49^. These findings corroborate our hypothesis that L-Cys selectively binds to the R-chiral gold surface and provides anisotropic deformation in the nanoparticles. It is expected that during the growth of the nanoparticles, L-Cys continuously attached mainly to the R region and inhibited the growth along the vertical direction, leading to lateral expansion of the R region, while the S region contracted; this asymmetric growth rate between the R and S regions resulted in the defined chiral structure of 432 helicoid IV. Inversely, in the case of D-Cys, we expect that as the molecule interacted with the S region, an exactly opposite 432 helicoid IV was synthesized. From the high-resolution transmission electron microscopy (HRTEM) analysis (Supplementary Fig. 7), high-index facets such as (331), (221), and (553) were observed, which indicates the interaction of Cys with a chiral high-index surface. Moreover, the asymmetric outlines of the elongated R region and contracted S region observed in the TEM (Supplementary Fig. 7) and SEM images (Fig. 2b) further support our hypothesis about the asymmetric growth of R and S regions in mechanism understanding.

### Growth pathways of 432 helicoid nanoparticles

**Fig. 3:**
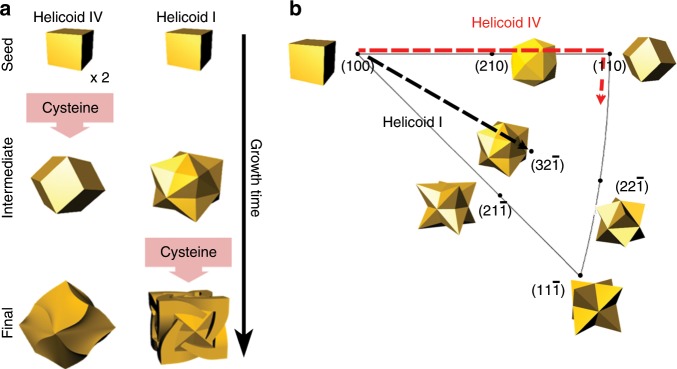
Growth pathway of 432 helicoid I and IV nanoparticles. **a** Schematic of morphological progression of helicoids, depending on time. From the top (initial) to the bottom (final), the sequential morphology changes are described with the synthesis conditions, the injection time of cysteine, and twice amount of seed in the case of 432 helicoid IV. **b** Growth pathway of helicoid nanoparticles plotted on a stereographic triangle that shows representative surfaces and morphologies. The black and red dashed line show pathway of 432 helicoid I and 432 helicoid IV, respectively.

We found that another key factor that determines the chirality evolution is the shape of the nanoparticles at an intermediate stage by comparing the case of 432 helicoids I and IV. Even though both nanoparticles utilize Cys to derive a chiral shape, substantially different morphologies are produced (Fig. 3a). The intermediate state of 432 helicoid I and IV show completely different shape of a stellated octahedron and RD, respectively. To achieve different basal shapes, different seed concentrations and injection times were used in each case. In the case of 432 helicoid I, a stellated octahedron shape was created by growing the cube seed for 20 min without involving the Cys molecule. For the 432 helicoid IV case, two times higher amount of seeds was added in the growth, which decreased growth kinetics of each gold nanoparticle and at the same time, the Cys molecule was involved from the beginning of the growth. As a result, RD shaped intermediates were obtained.

The difference in shape evolution between 432 helicoid I and IV was further understood by plotting the growth pathways of helicoids on stereographic projection of face centered cubic (FCC) crystal. The stereographic projection provides angular relation between surface facets, presenting all possible surfaces exposed in FCC structure. Based on SEM analysis of the time-dependent growth of nanoparticles, morphological change with time of all helicoid cases were illustrated on stereographic triangle (Fig. 3b; Supplementary Fig. 8). The growth pathways of 432 helicoid I and IV were plotted in stereographic projection with the crystallographic plane, and polyhedral shapes bounded by corresponding crystal planes (Fig. 3b). In the case of 432 helicoid I, cubic shape of seed continues to grow toward to the {321} index, which consisted of <100>, <110>, and <111> direction of growth and located at the center of the triangle. For the 432 helicoid IV case, however, the evolution of seeds progress along the edges of stereographic triangle, which follow the direction from (100) to (110). The increased amount of seed produces the reduced growth rate per seed in 432 helicoid IV growth condition, producing the RD intermediate of two low indices of (100) and (110) planes. In addition, while Cys seem to affect chiral growth after the development of {321} surface for the 432 helicoid I, the Cys for 432 helicoid IV case start to be involved even at the beginning of growth. The incorporation of Cys at these different crystallographic surface further affect chiral evolution. Then, the growth occurs along completely different pathway, resulting in significantly different final shapes (see Supplementary Fig. 8 and Supplementary Discussion 2 for a comparison of all types of helicoids).

From a geometrical aspect, the stellated octahedron and the RD are completely different polyhedrons since they are constructed by different combinations of concave and convex planes (Supplementary Fig. 9). In the stellated octahedron, the convex and concave angles of two adjacent R and S planes constitute the structure; specifically, A′B′ and A′C are convex boundaries, while B′C is concave (Supplementary Fig. 9a, b). The triangle A′B′C that corresponds to the R region is surrounded by three different triangular S regions, having A′B′, A′C, and B′C as the R–S boundaries. The convex angle between the R and S triangles connected by the A′C boundary can be clearly recognized by the red dashed line delineated in the 3D model (Supplementary Fig. 9b). The RD, on the other hand, is a convex polyhedron, and only A′B′ shows a convex angle, while B′C and A′C show coplanar alignment (Supplementary Fig. 9d, e). As observed in the 432 helicoid I and II cases, the shift in the R–S boundaries happens more dominantly in the convex than in the concave boundaries, and the reason for this phenomenon is still under investigation^38^. This factor suitably explains the different chiral structure evolutions in 432 helicoids I and IV. In the case of 432 helicoid IV, the chiral deformation is developed on a convex A′B′ boundary, and for 432 helicoid I, the chiral shift starts from the convex A′C boundary. These analyses suggest that the intermediate shape of the particle plays an important role in chiral morphology development by determining the location where the R–S boundary deformation occurs.

### Effect of Cys on chiral evolution

**Fig. 4:**
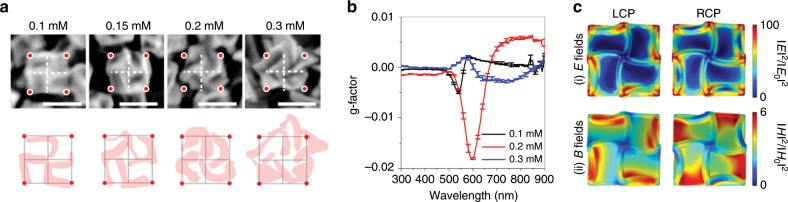
Effect of the cysteine concentration on the chiral morphology. **a** SEM images of synthesized nanoparticles under different cysteine concentrations ranging from 0.1 to 0.3 mM. The cysteine concentrations used in the synthesis are overlaid on the SEM images. The particles are aligned with the same <100> orientation. The centerlines (white dashed lines) and corners (red dots) of the nanoparticles are displayed in the SEM images to clearly show the deviation of the edges from the centers. The highlighted edges of each nanoparticle are represented at the bottom of the SEM images, and the edge deformation is directly compared based on the same black standard lines. Scale bars, 100 nm. **b** The g-factor spectra of nanoparticles prepared under different synthesis conditions of 0.1 (black), 0.2 (red), and 0.3 mM (blue) cysteine concentrations. Error bars are included in the spectra based on the statistical analysis over four different sample batches. Mean ± s.d. (*n* = 4) is shown. **c** Theoretical calculation of the electric (E, (i)) and magnetic field (B, (ii)) intensities of the 0.1 mM case of helicoid IV. Different field distributions are generated depending on the left circularly polarized (LCP) and right circularly polarized (RCP) light at 580 nm wavelength. Electric fields are expressed on a log scale, and magnetic fields are expressed on a linear scale.

Based on the understanding of the mechanism of chiral evolution, the effect of the Cys concentration on the chiral distortion was investigated. In Fig. 4a, nanoparticles synthesized with a range of Cys concentrations are shown with the same alignment of the <100> axis. To directly compare the degree of twist depending on the molecule concentration, the center and corners of the particle are drawn with white dashed crosses and red dots, respectively. In addition, the trace of chiral edges for each condition is separately displayed below the SEM image. For 0.1 mM Cys (the same condition as that of the nanoparticles shown in Figs. 1 and 2), the fourfold edge showed a translational shift from the central line. With an increase in Cys concentration up to 0.15 mM, the edge, the A′B′ boundary, continued to expand toward the S region, and this process generated bent edge that deviated from the central line. A substantial change in the edge was observed when the concentration exceeded 0.2 mM. The 0.2 mM Cys concentration led to significantly curved arms, and these highly curved edges showed a strongly enhanced CD spectrum, almost ten times larger than that observed for the 0.1 mM Cys concentration, generating the highest g-factor of 0.02 for 432 helicoid IV (Fig. 4b). The effect of curved edges of the nanoparticles on the generation of CD signals is also supported by the simulation results (Supplementary Fig. 10). The calculated CD and UV–Vis spectra of the helicoid IV model were consistent with the experimental results, and suggested that noticeable chiroptical property was originated from the high-order mode of plasmonic nanoparticles (Supplementary Fig.10). Figure 4c shows a near-field distribution of electric and magnetic fields at 580 nm in helicoid IV, depending on the left circularly polarized light and right circularly polarized light. From the different distribution of electric and magnetic moments generated by complex plasmonic chiral structures, we can expect that a strong CD response might be induced by the chiral distortion of edges. Quantitative analysis, depending on the deformation angle, will be discussed in a subsequent section.

Unlike the 0.2 mM case, the 0.3 mM Cys case showed a considerably decreased CD signal with overgrown edges. Due to this overgrowth, the uniformity of the chiral structure and axial symmetry in 432 helicoid IV (e.g., fourfold symmetry for the <100> direction) were significantly degenerated. In the case of the nanoparticles viewed along the <111> direction (Supplementary Fig. 11), a random direction of bending is observed due to the uneven growth of chiral edges that are connected to each other. These convoluted edges are expected to be the reason for the decreased CD signal. In addition, at high concentrations of Cys, the interaction of Cys with the metal surface get interfered, resulting in decreased enantioselectivity and the formation of random arms^38^. These arms can also be attributed to decreased CD intensity, which results in a broad spectrum.

Different concentrations of Cys can also affect the kinetics of chirality formation (Supplementary Fig. 12). In the case of 0.1 mM Cys, a discernable CD spectrum (−9.97 mdeg at 533 nm) was observed after only 20 min. However, for higher concentrations of Cys, the chirality was generated from an early stage of growth within 10 min: −17.77 mdeg at 540 nm for 0.2 mM and −41.59 mdeg at 563 nm for 0.3 mM. This increased kinetics in chiral formation is also observed in the morphological transition of nanoparticles over time. After 10 min of growth for the 0.2 mM case, a bent outline of an RD, which originated from the asymmetric growth of the R and S regions, was observed, where the shape corresponded to the 20 min growth time of 0.1 mM Cys. With the continuation of asymmetric growth, a curved edge with an increased bending angle was observed within 20 min. For the 0.3 mM case, highly twisted arms had already formed in 10 min due to expedited chiral formation, indicating accelerated growth kinetics with increased concentration of Cys. In addition, the peak shift and broadening of the resonant CD peak originating from a large chiral edge formation were accelerated with increasing Cys concentration. In the case of 0.1 mM Cys, a spectrum shift was rarely observed, and only an intensity increase occurred during the entire reaction. However, for 0.3 mM Cys, the resonance peak shifted to ~200 nm within 30 min due to large morphological deformation. With increasing Cys concentration, a broad spectrum was observed as a wide band of the spectrum, ranging from 600 to 700 nm for 0.2 mM Cys and from 600 to 800 nm for 0.3 mM Cys.

### Combinatorial effect of constituents on chiral development

**Fig. 5:**
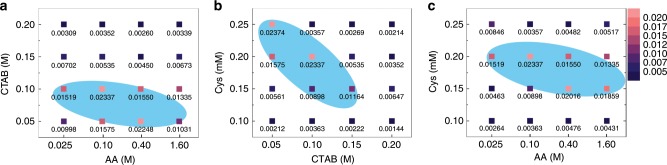
Effect of the various concentration of cysteine, CTAB, and AA on g-factor. **a**–**c** The g-factors of the synthesized nanoparticles under various concentration combinations of CTAB and AA (**a**), Cys and CTAB (**b**), and Cys and AA (**c**) were plotted. While the two variables changed, the other factor remained the same: **a** Cys 0.2 mM, **b** AA 0.1 M, **c** CTAB 0.1 M were fixed. The maximum g-factor (absolute value) of the synthesized nanoparticles under each condition is displayed with color and value. In the intensity scale on the left, pink represents the strongest and purple represents the weakest g-factor. High g-factor values above 0.01 are highlighted by blue area.

By adjusting the chemical constituents, Cys, CTAB, and AA in growth solution, chiral shape was controlled and the maximum g-factor was achieved (Fig. 5; Supplementary Fig. 13). Previously, we reported morphological control of gold nanoparticles based on the modulation of the effect of CTAB and AA on crystal growth^39^. In morphological control, CTAB generally provides flat surfaces in nanoparticle, since the Br^−^ ion specifically interacts on the surface of gold and the counter-ion, ammonium head with long alkyl chain, attaches on the Br^−^, making thick passivation on gold surfaces. In the case of AA, the increased concentration of AA leads to increase of gold ion reduction rate and growth kinetics, resulting in kinetically driven shape. Therefore, by balancing the influences of CTAB and AA, the growth along the different crystallographic direction and thus different crystal shape can be achieved.

Figure 5 shows combinatorial study to reveal how various CTAB-AA, Cys-CTAB, and Cys-AA concentration can influence on g-factor and structures. The corresponding CD and UV–Vis, and SEM images of synthesized structures are described in Supplementary Figs. 13, 14. When the concentration of CTAB and AA in the chiral growth solution was changed, while Cys concentration remains the same, different structural change was produced with drastic change of g-factor. The high g-factor were observed at low concentration of CTAB (below 0.1 M). From the SEM analysis, it is expected that the reason for decreased g-factor at high CTAB concentration is originated from the increased distance between chiral edges (Supplementary Fig. 14). The passivation effect of CTAB may result in the expansion of regions between chiral edges. As the CD response of chiral nanoparticle is directly correlated with the chiral edge, the distance change of chiral edges can significantly affect the chiral response. The g-factor comparison and SEM images of AA variation under the same CTAB and Cys condition shows that AA affect the sharpening of the chiral edge. As the chiral edge become prominent with AA, the increase of AA generates enhanced g-factor and provide high g-factor at wide range of concentration from 0.025 to 1.6 M. Based on the previous results, the effect of each chemical constituent, Cys, CTAB, and AA, can be summarized as follows; (1) cysteine increases bending degree of edge with the concentration increment, (2) CTAB enlarges distance between chiral edges due to increased passivation effect on the surface, and (3) AA heightens the chiral edges by increasing growth kinetics.

We further analyzed the relation between Cys with other two factors, CTAB and AA. Fig. 5b displays the correlative study of Cys and CTAB on g-factor. Under the low concentration of Cys (e.g. 0.1 mM), the g-factor is not significantly affected by the CTAB concentration due to small size and portion of chiral edges. As the chiral edge become large with increased curvature for high Cys concentration, change of distance of chiral edge which is influence of CTAB concentration greatly affect the g-factor. At high Cys concentrations where large chiral edges are developed with high curvature, the change in distance between chiral edges due to CTAB concentration greatly affects the g-factor, since the shape of the chiral edge is the key to CD signal generation. Therefore, the blue zone, highlighting the high g-factor, shows a steeply decreasing slope with increasing CTAB. In the high concentrations of CTAB and Cys, the decreased g-factor is due to large bending and wide edge distance, which deviates significantly from the optimal 432 helicoid IV structure with large g-factor. In Fig. 5c, the g-factor obtained at different concentration combination of Cys and AA is plotted. Unlike the effect of CTAB, the AA gives high g-factors over a relatively wide concentration range; the blue zone is distributed over a wide concentration from 0.025 to 1.6 M. Even at 0.15 mM Cys, an enhanced g-factor can be achieved by increasing the concentration of AA. Although the increase of AA offers advantageous effect on the enhancement of g-factor making prominent chiral edge, the increment range of g-factor is relatively small compared with Cys effect. This trend suggests that Cys is more dominant factor in g-factor enhancement than AA.

### Theoretical calculation on chiral structure in helicoid nanoparticle

To gain a more quantitative understanding of the relationship between the chiral structure and optical response, we further performed numerical simulations on various structures achieved by synthetic modulation (Supplementary Fig. 15). As the growth proceeds, modifications, such as bending, protrusion, and widening, mainly occur on the edge of the nanoparticle, and the degree of these changes is directly related to the g-factor of the helicoid nanoparticle. From the SEM analysis, we constructed models that describe the unique structural parameters of the chiral nanoparticles. Detailed descriptions of the models and simulations are provided in the Supplementary Discussion.

The bending degree of the chiral edge was evaluated for the chiral response (see Supplementary Fig. 15a for the definition of the bending angle *ϕ*). By increasing the angle of *ϕ*, a highly twisted chiral edge was introduced, resulting in an enhanced CD response. For example, as the angle increased from 6° to 22°, a threefold increase in the g-factor was observed. On the other hand, an extreme bending angle at 27° significantly reduced the CD intensity with a broad spectrum, possibly originating from the changed chiral field distribution by the close opposite side of the edges. In the case of protrusion of the edge, a significantly enhanced chiral response was observed, depending on the pronounced amount (Supplementary Fig. 15e). As the height of protrusion was increased by factors of 2 and 4, fourfold and tenfold increments of the g-factor were observed, respectively. The enhancement of the g-factor by protrusion showed the most significant effect compared with that of other factors, such as the size, bending degree, and width of the edge. An effect of the width of the edge on the optical response was simulated using two models that describe the modification of the width in different ways (Supplementary Fig. 15f, g). In both models, an increase in the CD intensity was clearly observed, depending on the enhancement of the width. However, when the widened edges were in close proximity, that is, close enough to make coupling, the g-factor was significantly decreased, and a different broad spectrum was observed. This spectral change was also observed in the largely bent chiral edge case, which indicates that different coupling modes between the chiral components lead to changes in the optical response. Finally, as shown in the previous results, the interplay of structure factors provides a synergistic effect and strong g-factor enhancement. This result suggests that depending on the combination and ratio of key structural factors, we can elicit a new optical response. Interestingly, when we increased the effect of the protrusion and width of the edge simultaneously, substantially different spectral features with very strong g-factor were simulated (Supplementary Fig. 15h #32). A combinatorial study of the key structure parameters for a large g-factor along with the experimental demonstration of the structure is still under investigation.

### Film-type of assembled helicoid nanoparticle

**Fig. 6:**
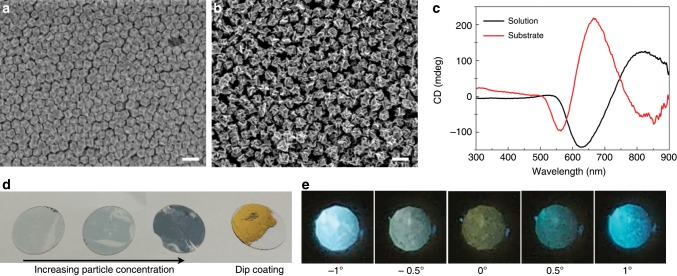
Morphology and optical characteristic of the substrates coated with 432 helicoid IV. **a**, **b** Large-area SEM image of the substrate coated with nanoparticles by the dip-coating method. Two different types of 432 helicoid IV, made with 0.1 mM (**a**) and 0.2 mM (**b**) of Cys concentration, were used for coating. A monolayer or bilayer of assembled nanoparticles was uniformly generated on the substrate. **c** CD spectra of nanoparticles in the solution phase (black) and coated on the substrate (red). The coupling of assembled nanoparticles in the film-type results in a significant change in spectral features compared with the results of the nanoparticle solution. **d** Photograph of 432 helicoid IV assembled substrates with different concentrations of nanoparticles. For the dip-coating sample, which has the highest packing density, the gold reflection color is shown. **e** Polarization-resolved transmission image of a substrate coated with nanoparticles. The angle of the analyzer was changed from −1° to 1°. See the Methods section for coating procedures and polarization experiments. Scale bars, 200 nm.

One of the advantages of the 432 helicoid IV structure lies in its ability to assemble. The unique basal shape of the rhombic dodecahedron enables high-density packing of 432 helicoid IV nanoparticles. Figure 6a, b show the film-type assembled helicoid nanoparticles fabricated by the method of dip coating onto a glass substrate. Two or three layers of nanoparticles were closely assembled with a hexagonal pattern. Interestingly, a significant change in the spectrum, blueshifting, and a different ratio of the bisignate peak were observed in the substrate case due to strong coupling between closely assembled particles (Fig. 6c). The high density and strong coupling of 432 helicoid IV resulted in a strong reflective color, as shown in Fig. 6d, corresponding to the dip-coating case. In addition, by utilizing the electrostatic coating method, the amount of nanoparticles on the substrate was changed (Supplementary Fig. 16a–c). Increasing the density of helicoid nanoparticles resulted in a gradual CD spectral shift originating from the coupling between particles and an increase in the blue scattering color. These results present a new direction for achieving a chiroptical response by utilizing various assembly techniques that can control the coupling of nanoparticles.

Next, a macroscopic color change was observed by placing the film-type helicoid substrate between the cross-polarized filters and rotating the analyzer. Under the cross-polarized conditions of the polarizers, an olive transmitted color was generated, which is different from the achiral case (Fig. 6e; Supplementary Fig. 16d, e). When the analyzer was rotated to counterclockwise (toward 1°), the blue and green hues were increased, resulting in cyan transmittance, and for clockwise rotation (towards 1°), a transition to a sky-blue color was generated. As the interaction between the circularly polarized light and the helicoid particles was different depending on the wavelength, an asymmetrical color change was observed with the angle of the analyzer, while the achiral sample showed only a symmetric color change. The successful transfer from the solution phase to a film-type substrate suggests that this film-type helicoid nanostructure can be directly utilized to form optical components such as chiral polarizers and to enable many other applications. Furthermore, a complex asymmetric metamaterial can be created by combining this helicoid substrate with conventional thin film deposition techniques.

In summary, we studied chirality formation in a nanoparticle with a focus on growth environment control. In particular, as an extension of a previous study on amino acid and peptide-induced chirality in a nanoparticle, we investigated the diverse components that can affect chiral development. The Cys molecule plays an important role in determining the handedness, level of bending, and kinetics of chirality development. The morphology of the intermediate seed is also a critical factor in chiral structure evolution, because the angle between the R and S planes determines the starting point of chiral deformation. CTAB and AA also play essential roles in terms of changing the dimensions of chiral edges. Based on the mechanism understanding of the growth condition, the chiral structure of 432 helicoid IV was grown, which resulted in a strong g-factor. Using the advantages of 432 helicoid IV, we have shown different optical responses by assembling nanoparticles. By fabricating film-type helicoid nanoparticles, the applicability of helicoids was demonstrated. The enhanced understanding of chirality development and its application will be beneficial in fabricating diverse chiral plasmonic nanostructures and will bring about possibilities for unprecedented morphologies and functions.

## Methods

### Chemicals

Hexadecyltrimethylammonium bromide (CTAB, 99%), L-ascorbic acid (AA, 99%), tetrachloroauric (III) trihydrate (HAuCl_4_∙3H_2_O, 99.9%), 1-dodecanethiol (≥98%), and sodium borohydride (NaBH_4_, 99%) were purchased from Sigma-Aldrich. n-Hexane (85%) and absolute ethanol were purchased from Daejung Chemicals & Metals. L-cysteine hydrochloride monohydrate (99%, TCI) and D-cysteine hydrochloride monohydrate (99%, TCI) were used without further purification. All aqueous solutions were prepared using high-purity deionized water (18.2 MΩ/cm).

### Synthesis of the cuboctahedron seeds

First, small 2-nm-sized spherical nanoparticles were synthesized by the rapid addition of a strong reducing agent, NaBH_4_ (10 mM, 0.8 mL), into a CTAB (100 mM, 7.5 mL)- and HAuCl_4_ (10 mM, 0.25 mL)-containing solution. The solution was left for 3 h to decompose the NaBH_4_. The 1/10 diluted spherical seeds were grown in the growth solution, which contained water (8 mL), HAuCl_4_ (10 mM, 0.2 mL), CTAB (100 mM, 1.6 mL), and AA (50 mM, 0.95 mL). After 15 min, the solution turned pink, which indicated the formation of 50-nm-sized cuboctahedron nanoparticles. The resultant crystals were washed twice by centrifugation (6708 *g*, 150 s) and redispersed in a 1 mM CTAB solution.

### Synthesis of 432 helicoid IV

To synthesize chiral nanoparticles, the growth solution was prepared by mixing water (3.95 mL), CTAB (100 mM, 0.8 mL), HAuCl_4_ (10 mM, 0.1 mL), AA (0.1 M, 0.475 mL), and cysteine (0.1 mM, 5 μL). With the addition of premade cuboctahedron nanoparticles (100 μL), the growth of chiral nanoparticles was started and continued for 1 h. The solution was kept in a 30 °C bath during the reaction to prevent the crystallization of CTAB. The pink solution gradually turned blue with a large scattering color. To remove the unreacted chemicals, the solution was centrifuged twice and stored in a 1 mM CTAB solution.

### Preparation of film-type helicoid nanoparticles

Dip-coating method: First, 1 mL of a suspension of 432 helicoid IV nanoparticles and 1 mL of deionized water were mixed and prepared in a 10 mL glass vial. Two milliliters of n-hexane solution was loaded on top of the prepared solution. Five microliters of 1-dodecanethiol was carefully dropped on the liquid/liquid interface. After 1 min, 5 mL of absolute ethanol was injected into the helicoid nanoparticle solution. After the injection of absolute ethanol, a monolayer of helicoid nanoparticles was formed in the interface with the color of bulk gold. Next, n-hexane was carefully removed from the top of the solution. Finally, the monolayer of helicoid nanoparticles was transferred to a glass substrate.

Electrostatic coating method: 200 μL of 432 helicoid IV nanoparticles (suspended in 1 mM CTAB solution) was washed twice with deionized water to produce helicoid nanoparticles suspended in 5 μM CTAB solution. During nanoparticle washing, glass substrates were treated with plasma (200 W) for 5 min. Two hundred microliters of the prepared helicoid nanoparticles were dropped onto plasma-treated glass substrates and left for 1 h. The glass substrates coated with helicoid nanoparticles were washed with deionized water and dried by blowing N_2_ gas. The density of the nanoparticles on the glass substrate was controlled by changing the concentration of helicoid nanoparticles in solution.

### Polarization-resolved color generation

The polarization-rotating ability of the 432 helicoid IV substrates was visualized by a cross-polarized optical configuration: a white-light source, vertical aligned linear polarizer, film-type of helicoid IV, horizontal aligned linear polarizer (analyzer), and digital camera. Substrates prepared through the dip-coating method or electrostatic coating method were placed between two linear polarizers with a cross-polarized state (0° represents cross-polarized conditions). The analyzer was rotated from −1° (clockwise) to 1° (anticlockwise) at 0.2° intervals from the orthogonal configuration. The macroscopic color change of transmitted light during rotation of the analyzer was observed and recorded through a digital camera.

### Characterization

SEM images were taken using a SIGMA system (Zeiss, Oberkochen, Germany). To obtain transmission electron microscopy (TEM) images, a JEM-3000F system (JEOL, Tokyo, Japan) was used. A J-815 spectropolarimeter (JASCO, Tokyo, Japan) was used for extinction and CD measurements. Kuhn’s dissymmetry factor (g-factor), which shows the degree of chirality, was calculated from the extinction and CD values by the following equation.

$${\mathrm{g}} {\hbox{-}} {\mathrm{factor}} = 2\frac{{A_{\mathrm{L}} - A_{\mathrm{R}}}}{{A_{\mathrm{L}} + A_{\mathrm{R}}}} \propto \frac{{{\mathrm{CD}}}}{{{\mathrm{extinction}}}}$$

### Numerical calculations

The 432 helicoid IV nanoparticles were quantitatively analyzed through numerical full-wave electrodynamics simulation using FEM simulation (COMSOL Multiphysics 5.4). Several helicoid IV models based on the SEM images showed similar spectral features consistent with the experimentally measured spectra (see Supplementary Discussion 3 and Supplementary Fig. 10). Here, extinction = (σ_+_ + σ_−_)/2, CD = σ_+_ − σ_−_, and g-factor = CD/extinction, where σ_±_ is the extinction cross sections of the nanoparticles by LCP/RCP (±) light propagating in the +*z* direction. The orientation-averaged extinction of small nanoparticles was approximated as 〈σ〉 = (σ_+*x*_ + σ_−*x*_ + σ_+*y*_ + σ_−*y*_ + σ_+*z*_ + σ_−*z*_)/6, where ±*x*, *y*, and *z* indicate the propagation direction of the incident planewave^50^. The chiral nanoparticle used in this work has fourfold rotational symmetry, such that σ_+*x*_ = σ_−*x*_ = σ_+*y*_ = σ_−*y*_ = σ_+*z*_ = σ_−*z*_, so we considered only σ_+*z*_ = σ for the calculations. The extinction and CD at different orientations are nearly identical (Supplementary Fig. 10), although small deviations are observed at ~600–650 nm, where a higher-order multipole contribution exists. The electromagnetic responses of an isolated nanoparticle in a homogeneous medium were implemented by using a scattered-field formalism with a background field given by a circularly polarized planewave and a perfectly matched layer surrounding the system. The mesh was constrained to be smaller than the feature size of the nanoparticles inside the nanoparticle, and smaller than *λ*/7 for the rest of the system. The optical constants of gold were taken from Johnson and Christy^51^, and the host medium was water with a refractive index of 1.33.

## Supplementary information

Supplementary Information

## Data Availability

The data within this paper and other finding of this study are available from the corresponding author upon reasonable request.
