# Supplementary material for: Cysteine-encoded chirality evolution in plasmonic rhombic dodecahedral gold nanoparticles
Source: Nat Commun. 2020 Jan 14;11:263. doi: 10.1038/s41467-019-14117-x (PMC6959252; doi:10.1038/s41467-019-14117-x)
Supplement: Supplementary file 1 — Supplementary Information [file 41467_2019_14117_MOESM1_ESM.pdf]

Supplementary Information

Cysteine-Encoded Chirality Evolution in Plasmonic Rhombic Dodecahedral Gold Nanoparticles

*Hye-Eun Lee[1], Ryeong Myeong Kim[1], Hyo-Yong Ahn[1], Yoon Young Lee[1], Gi Hyun Byun[1], Sang Won Im[1], Jungho Mun[2], Junsuk Rho[2,3] and Ki Tae Nam[1]**

[1]Department of Materials Science and Engineering, Seoul National University, Seoul 08826, Korea

[2]Department of Chemical Engineering, Pohang University of Science and Technology (POSTECH), Pohang 37673, Korea

[3]Department of Mechanical Engineering, Pohang University of Science and Technology (POSTECH), Pohang 37673, Korea

This file includes:

Supplementary Figures (Figure 1–16)

Supplementary Discussion 1–3

References

* To whom correspondence should be addressed:

Ki Tae Nam, Ph.D.

Department of Materials Science and Engineering,

Seoul National University

Supplementary Figures

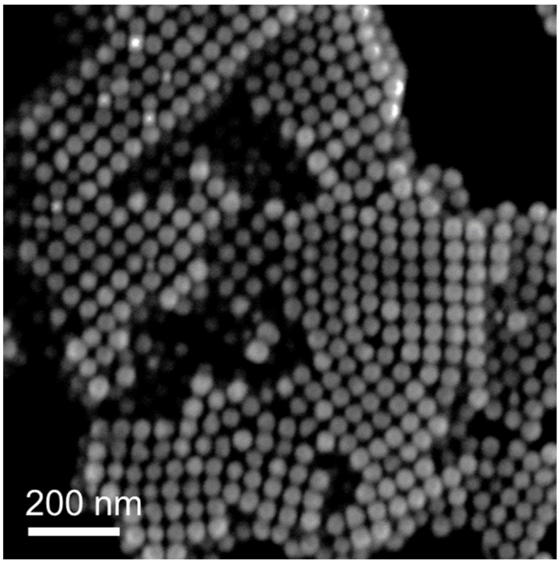

Supplementary Figure 1. SEM image of cuboctahedron seeds.

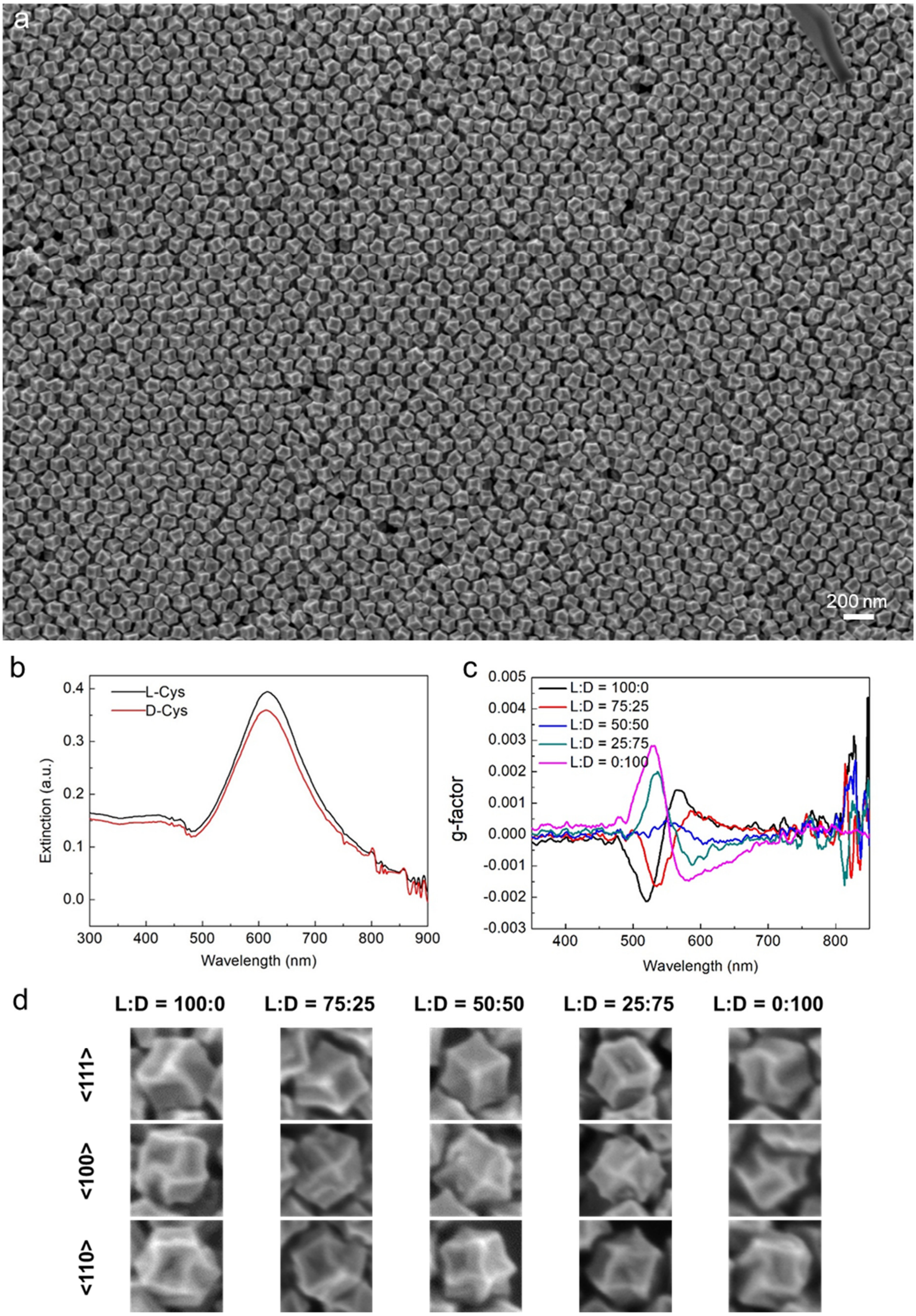

Supplementary Figure 2. Characterization of 432 helicoid IV. (a) Large-area SEM image of 432 helicoid IV. The

yield of one handedness of chiral nanoparticle was 94.76 % of a total of 1963 nanoparticles. (b) Extinction spectra of 432 helicoid IV synthesized using L-Cys (black) and D-Cys (red). (c) g-factor spectra of nanoparticle synthesized under different mixture ratio of L-Cys : D-Cys. The black, red, blue, green and pink lines correspond to the 100 : 0, 75 : 25, 50 : 50, 25 : 75, 0 : 100 ratios of L-Cys : D-Cys, respectively. (d) SEM images of the resultant nanoparticles synthesized under different mixture ratios of L-Cys : D-Cys, viewed along the <110>, <111>, and <100> axes.

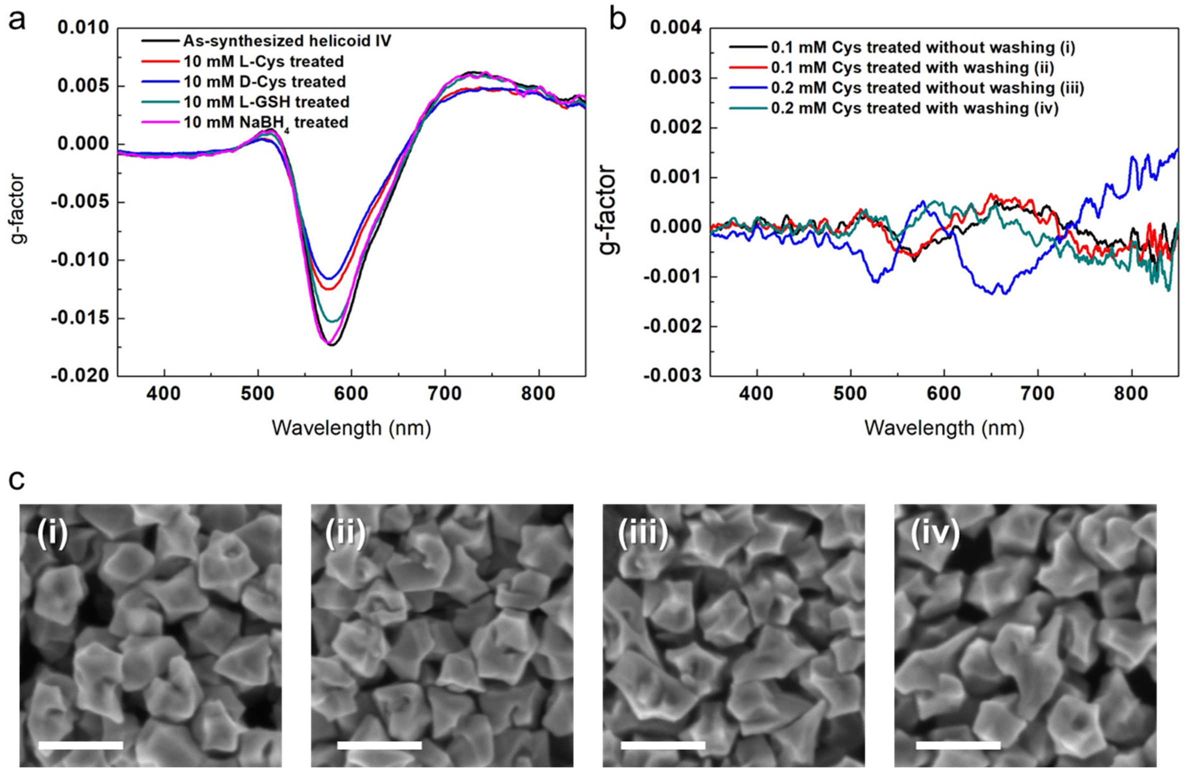

Supplementary Figure 3. Effect of chiral ligands on chiroptical response. (a) Comparison of g-factor spectra after attaching and detaching chiral ligands. As-prepared 432 helicoid IV nanoparticle was shown in black line (no further treatment). Three different type of chiral ligands, L-Cys (red line), D-Cys (blue line), and L-glutathione (GSH, green line), were further attached on the as-prepared 432 helicoid IV. For detachment of chiral ligand in 432 helicoid IV, $NaBH_4$ was treated (pink line). (b, c) Effect of pre-adsorbed Cys molecule on the chirality of nanoparticle. The g-factor spectra (b) and SEM images (c) of resultant crystal synthesized using different pre-adsorbed Cys conditions of the seed. Two different concentrations, 0.1 mM (i, ii) and 0.2 mM (iii, iv), of Cys were first added to the seed nanoparticles and one sample was prepared without washing (i, iii) and the other was prepared with washing to get rid of remaining Cys (ii, iv). The Cys adsorbed seed nanoparticles further grown in growth solution without containing Cys molecules. All scale bars are 200 nm. For a detailed explanation, see Supplementary Discussion 1.

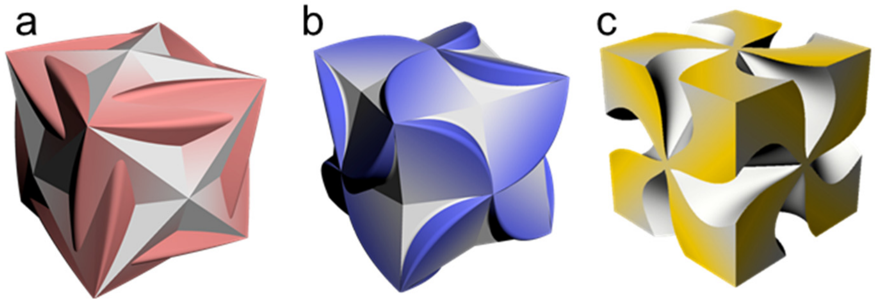

Supplementary Figure 4. Schematic of 432 helicoid I (a), II (b), and III (c) reported in the previous work.

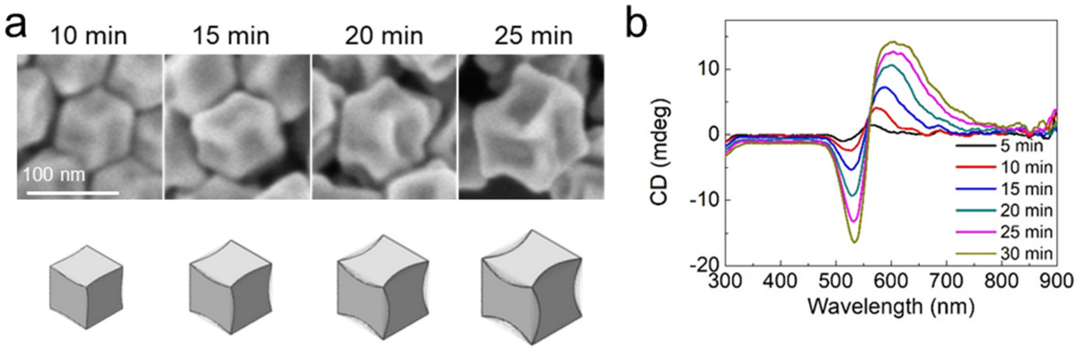

Supplementary Figure 5. Morphological development of 432 helicoid IV depending on growth time. SEM images with corresponding model (a) and CD spectra (b) of nanoparticles.

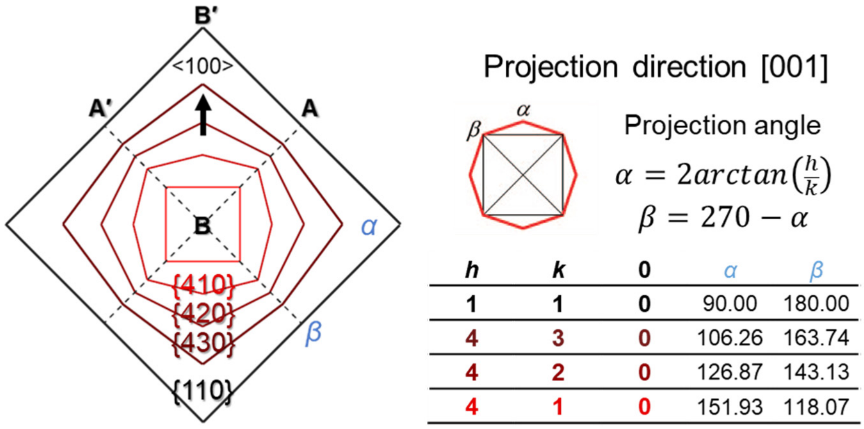

Supplementary Figure 6. Projection models along <001> direction with calculated Miller-indices, {hk0}, and the equation that correlates the index and projection angle of nanoparticles. The same rhombus ABA′B′ indication that is shown in Figure 2a is also shown in the scheme. Note that during the transformation from cube to RD via <100> growth, diverse high-Miller-index facets are generated on the nanoparticle surface.

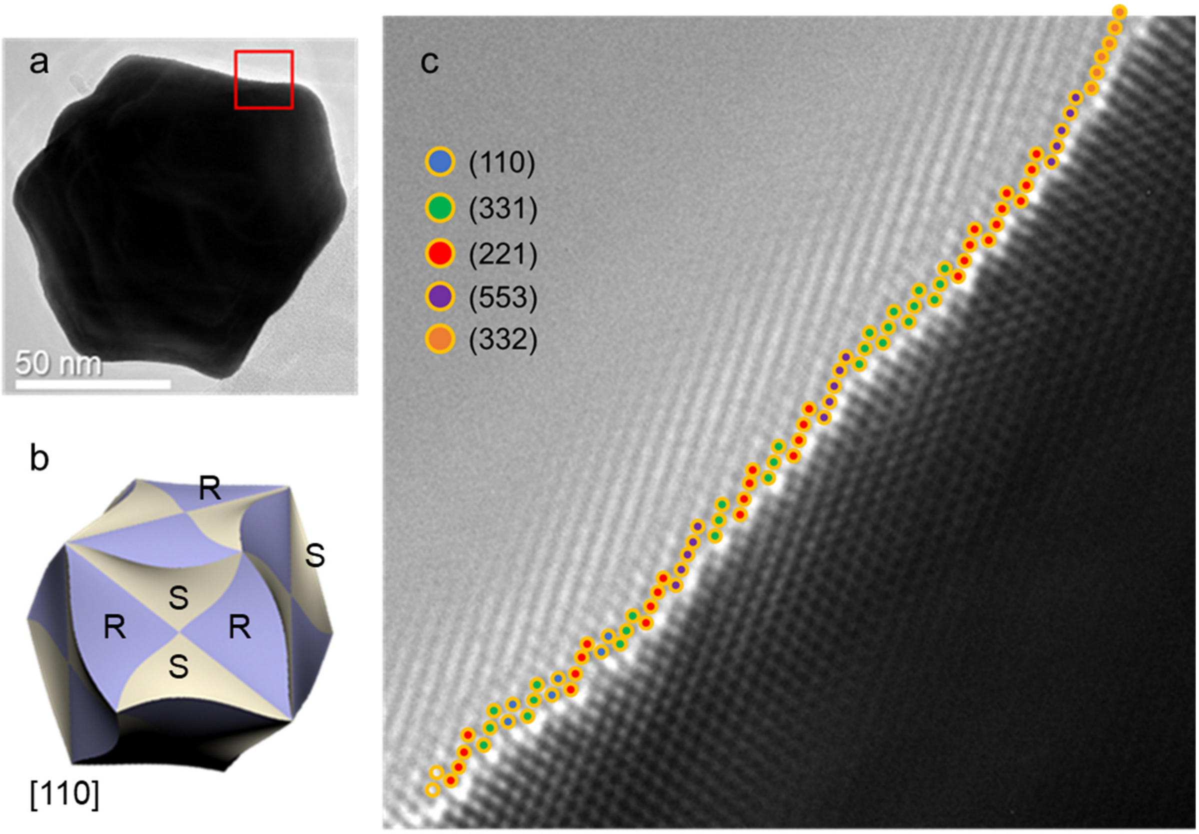

Supplementary Figure 7. TEM analysis of 432 helicoid IV. (a) TEM image of chiral nanoparticle viewed along the [110] direction. Corresponding chiral scheme is shown in (b) with indication of R and S regions. The sample was prepared after 20 min of particle growth, where chiral distortion is observed due to the attachment of cysteine. Based on the R and S region information in (b), we can assume that elongated edge and decreased edge observed in the top-right side of the nanoparticle in (a) originated from the expansion of R region and contraction of S region, respectively. (c) HRTEM image showing detailed atomic arrangement in the area enclosed in red rectangle in (a). Each atom is marked with a colored sphere and different colors represent different indices of the microfacets. From the indications of microfacets shown in different colors in the HRTEM image, various high-index exposed surfaces are identified.

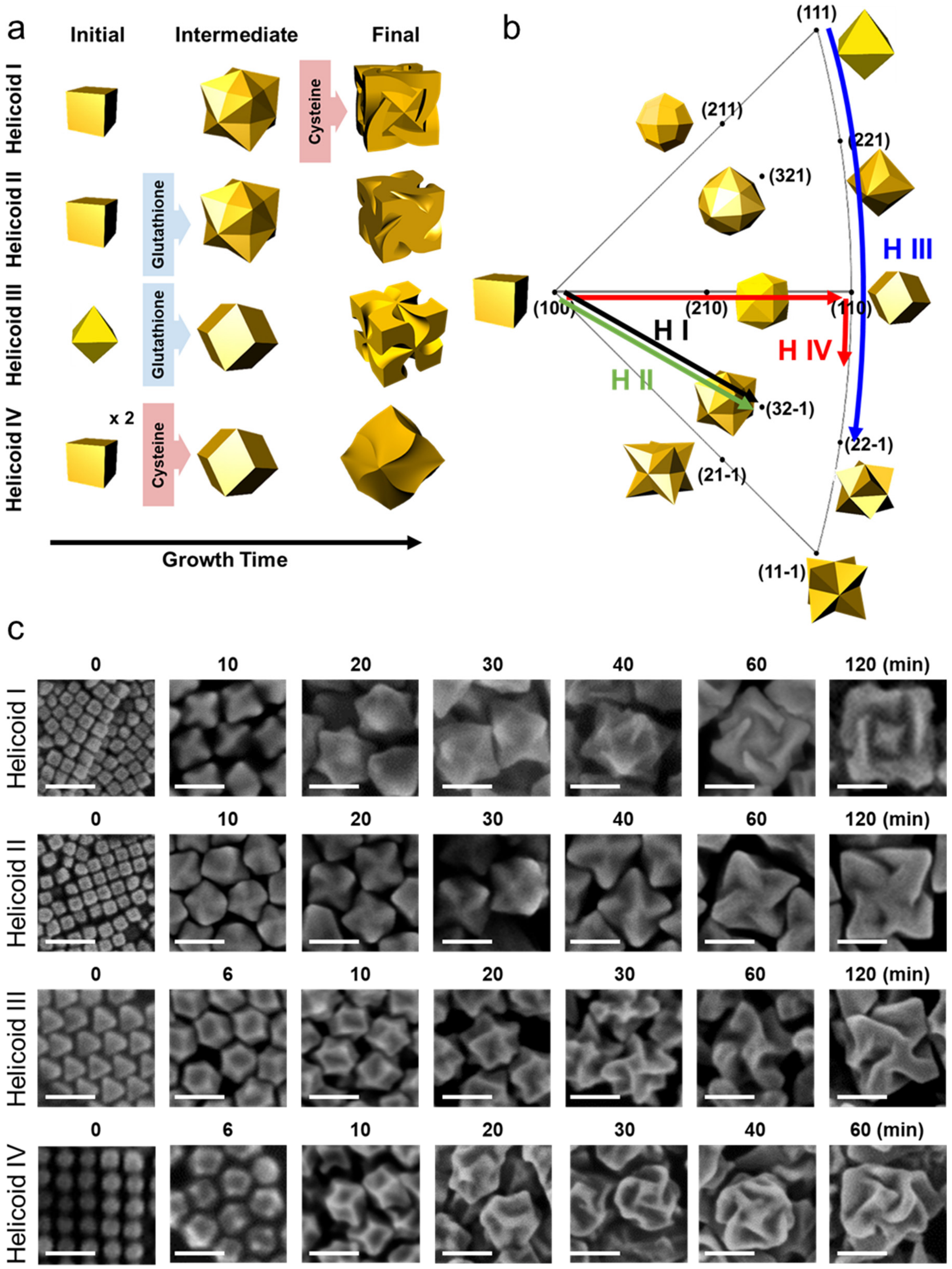

Supplementary Figure 8. Growth pathway of 432 helicoid I, II, III and IV nanoparticles. (a) Schematic of morphological evolution of helicoids depending on time. From the left (initial) to the right (final), the shape changes of helicoid nanoparticles are described with each synthesis condition, type of seed, injection time of chiral ligand (cysteine and glutathione), and amount of seed. (b) Growth pathways of all helicoid nanoparticles

plotted on a stereographic projection. For a clear visualization of the different shape evolution of each helicoid case, the crystallographic planes and polyhedral shapes bounded by the corresponding crystal planes are illustrated. Black, green, blue, and red lines show growth pathway of 432 helicoid I, II, III and IV, respectively. (c) SEM images of helicoids I, II, III, and IV at different growth times. For further explanation, see Supplementary Discussion 2. All scale bars are 100 nm.

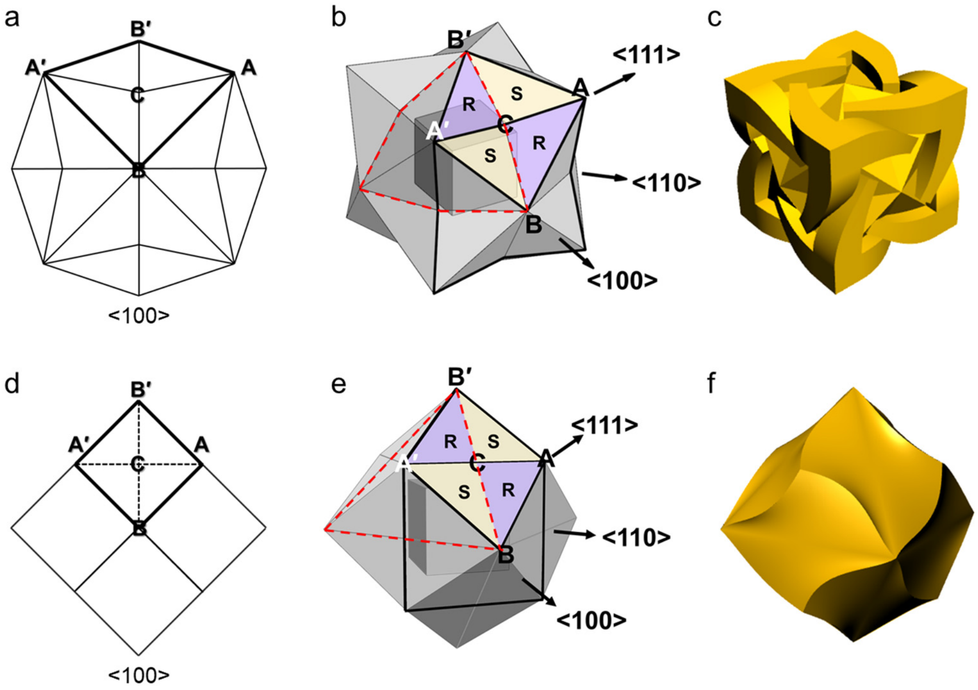

Supplementary Figure 9. Geometrical analysis of initial stages of nanoparticle for 432 helicoid I and IV. Schematic illustrations of 432 helicoid I (a–c) and 432 helicoid IV (d–f) show initial stages of growth ((a,d) for <100> projection and (b,e) for three-dimensional model) and final shapes (c,f). Representative rhombus ABA′B′, and R and S chiral conformations of gold are illustrated in the initial models. For clear visualization of angle between R and S planes, BC and B′C boundaries are highlighted with red dashed lines (b,e).

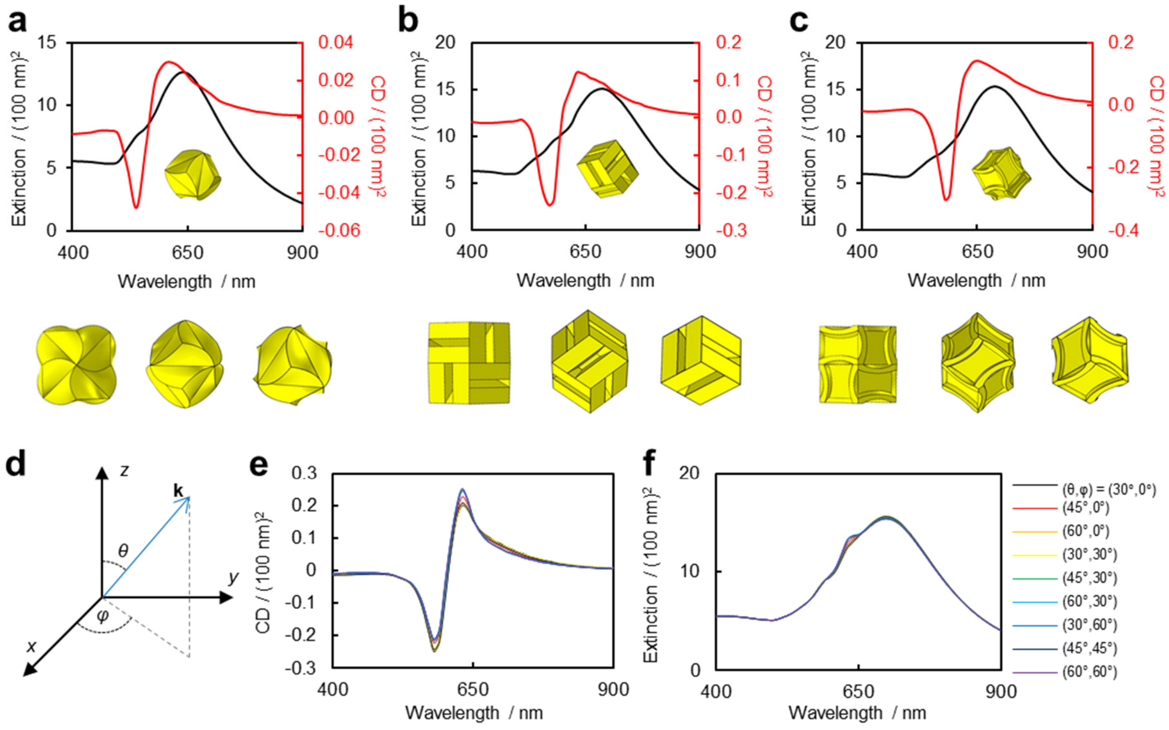

Supplementary Figure 10. Numerical simulation results of the 432 helicoid IV model. (a–c) Calculated extinction (black) and CD (red) spectra for 432 helicoid IV with different models based on SEM images. All three models are constructed on identical rhombic dodecahedra. Based on the RD shape, different alterations were applied to describe the chiral twist. The corresponding three-dimensional models are displayed below the spectra. All three models successfully reproduced the characteristic experimental spectrum peak; two main absorbance peaks, 'bisignate' CD signals and CD peaks overlapped on the increased extinction spectrum. The extinction and CD were calculated at normal incidence taking advantage of the small size and 4-fold rotational symmetry. (d–f) Calculated CD (e) and extinction (f) at different propagation directions of the incident planewave.

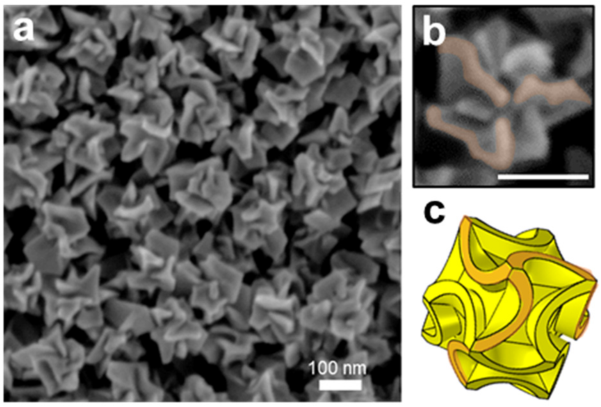

Supplementary Figure 11. SEM image of nanoparticles synthesized under 0.3 mM Cys. (a) Large-area SEM image of nanoparticles showing diverse shapes of the chiral arm. (b) Enlarged image of a nanoparticle viewing along the (111) direction. (c) Schematic model of 432 helicoid IV with a high degree of edge twist. In comparison with the schematic model and enlarged image of 432 helicoid IV in the (111) direction, several broken chiral arms that would normally be connected are observed in the SEM image, corresponding to reduced chirality in this nanoparticle. All scale bars are 100 nm.

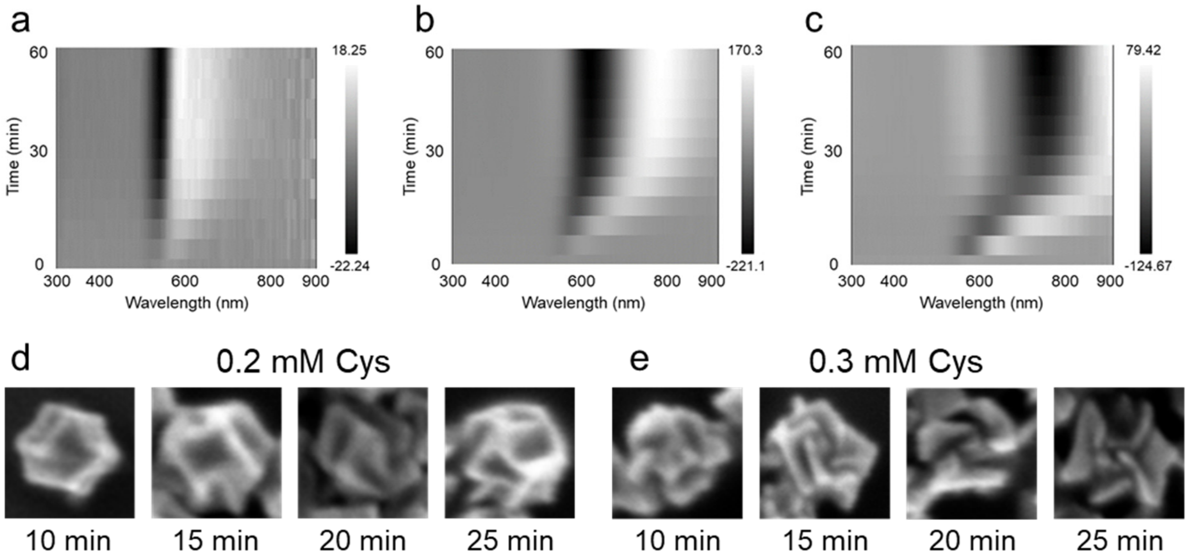

Supplementary Figure 12. Temporal evolution of the CD spectrum of 432 helicoid IV nanoparticles depending on the cysteine concentration: (a) 0.1 mM, (b) 0.2 mM, and (c) 0.3 mM. The CD spectra were measured at 5 min intervals. The measurement was started from 5 min after injection of the seed and continued until 60 min of growth. The spectra are displayed as a function of wavelength and time. The intensity of the CD spectrum is represented via black-and-white contrast. The color scale is shown on the right side of the spectrum. (d, e) Morphological development of 432 helicoid IV under 0.2 mM Cys (d) and 0.3 mM Cys (e) depending on the growth time.

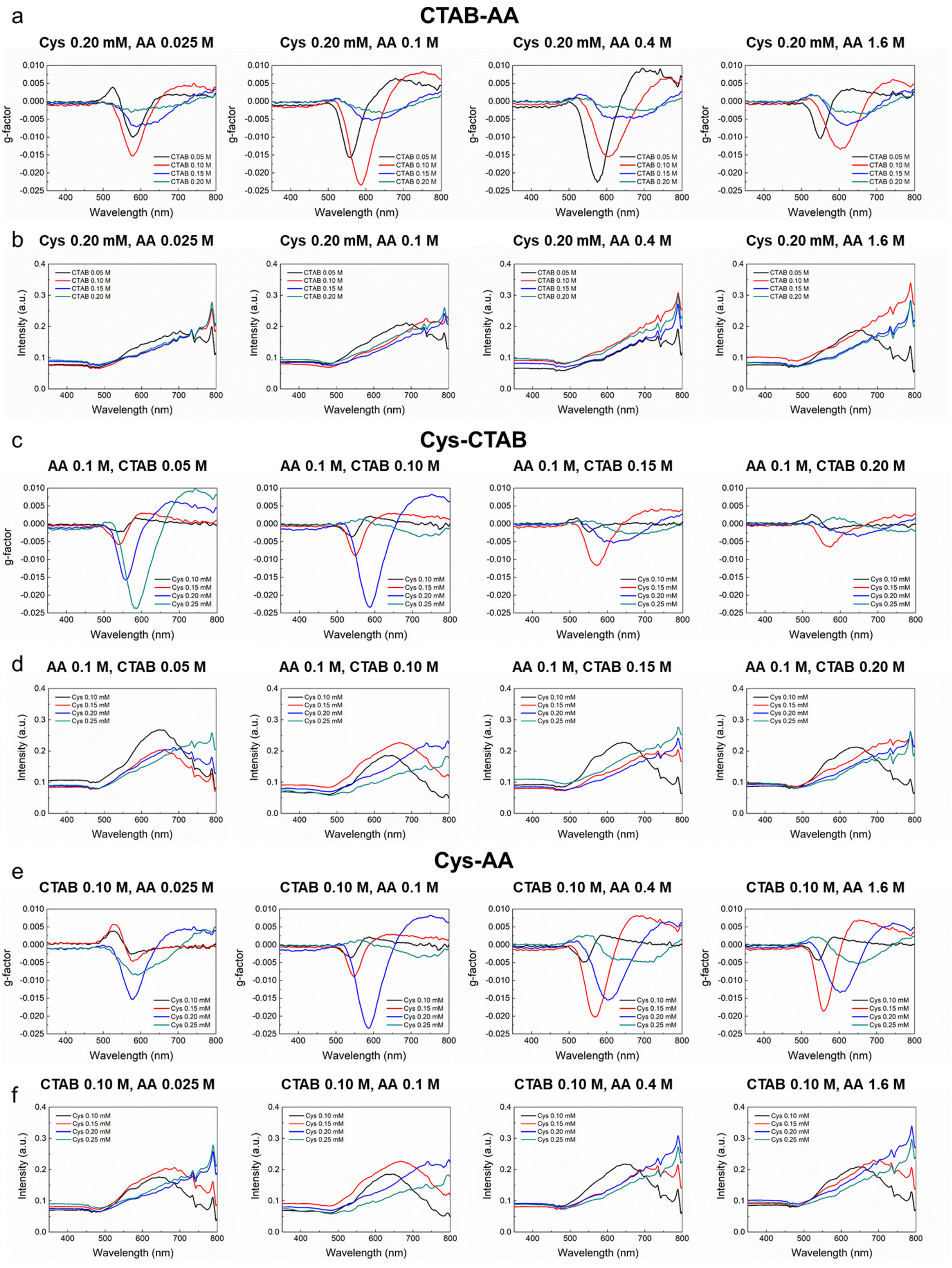

Supplementary Figure 13. Comparison of the g-factor and UV-Vis spectra of nanoparticles prepared under diverse synthesis conditions: (a, b) Effect of CTAB and AA concentration changes at 0.2 mM Cys fixed concentration. (c, d) Effect of Cys and CTAB concentration changes at 0.1 M AA fixed concentration. (e, f) Effect of Cys and AA concentration changes at 0.1 M CTAB fixed concentration. The g-factor (a, c, e) and UV-

Vis (b, d, f) spectra of the synthesized nanoparticle are plotted with each synthesis condition.

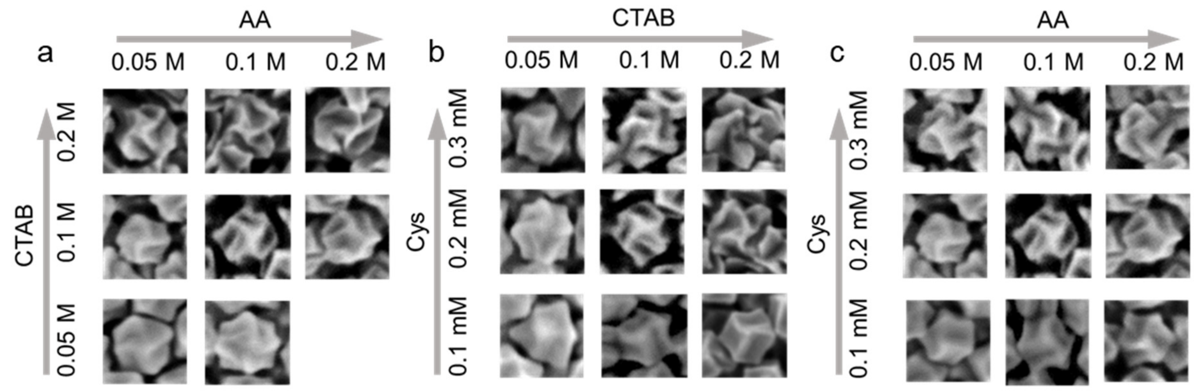

Supplementary Figure 14. SEM images of nanoparticles prepared under diverse synthesis conditions: (a) CTAB and AA concentration variables with fixed 0.2 mM Cys, (b) Cys and CTAB concentration variables with fixed 0.1 M AA, (c) Cys and AA concentration variables with fixed 0.1 M CTAB.

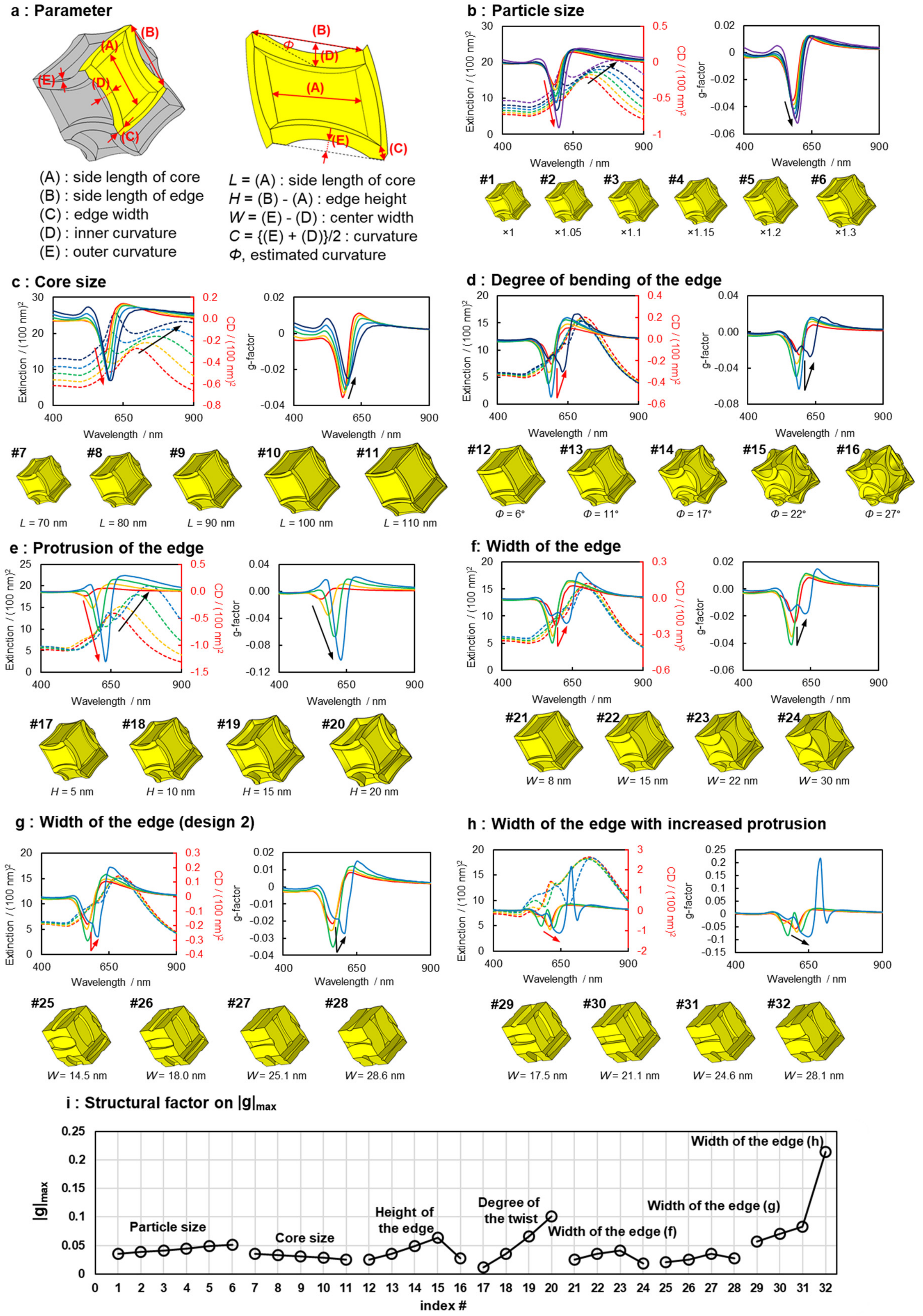

Supplementary Figure 15. Simulation results of structural modification in 432 helicoid IV. (a) Definition of the parameter for modification. (b–h) Calculated extinction (dashed line, left), CD (solid line, left), and g-factor (solid

line, right) spectra of gold chiral nanoparticles with corresponding models. Different structural parameters were studied: the particle size (b, #1–6); core size (c, #7–11); degree of bending of the edge (d, #12–16); protrusion of the edge (e, #17–20); width of the edge (f, #21–24); width of the edge (design 2) (g, #25–28); and width of the edge with increased protrusion (h, #29–32). The trend of the transition spectra from a small number to a large number of models is also indicated by the direction of the arrow. (i) Comparison of the g-factors of the chiral nanostructures studied above.

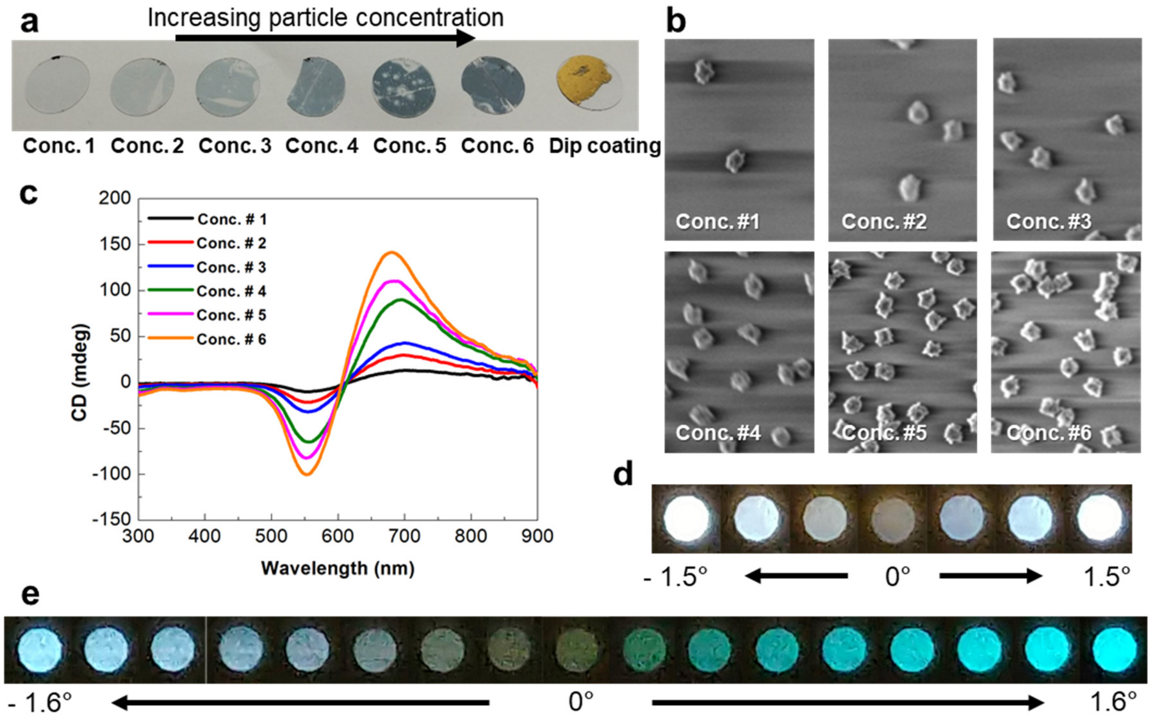

Supplementary Figure 16. Optical properties of the substrate coated with 432 helicoid IV nanoparticles. (a-c) Density dependence on the optical properties of the nanoparticle-coated substrate. (a) Photograph of a glass substrate coated with different concentrations of 432 helicoid IV nanoparticles. The plasma-treated coating process was used to vary the attached amount of nanoparticles on the substrate (see Methods). An increasing color contrast was observed with increasing nanoparticle density. A reflective gold color was generated for the case of the substrate treated with the dip-coating procedure. (b) SEM images showing the particle density of the substrate prepared in (a). (c) CD spectra of the substrate with different particle densities. With increasing density, the two peaks with different ratios from the solution case gradually increase due to the coupling between the particles. (d, e) Polarization-resolved transmission images of a substrate coated with achiral nanoparticles (d) and 432 helicoid IV (e). Starting from the cross-polarized condition (0°), the angle of the analyzer was changed from -1.5° to 1.5° in the case of the achiral sample and -1.6° to 1.6° in the case of the 432 helicoid IV sample. The helicoid substrate shows an asymmetrical color transition depending on the angle, while the achiral sample displays only a symmetrical change.

Supplementary Discussion 1

Chiroptical response in plasmonic nanostructure can be generated by several mechanisms. Generally, the mechanism of CD signal generation can be categorized by 1) plasmon-induced chirality, 2) intrinsically chiral nanoparticle structure, and 3) nanoparticle assembly with handed configurations[1,2]. In order to identify which mechanism dominates the chiral response of 432 helicoid IV, we performed series of control experiments (Supplementary Figure 3). The first case can be obtained by incorporation of chiral organic molecules to the plasmonic structure. Therefore, here we conducted and analyzed the additional experiment by focusing on the organic ligand of the 432 helicoid IV surface. For experimental demonstration of plasmonic-induced chirality, two methods, attaching chiral ligand[3,4] and embedding chiral molecule in plasmonic structure[5-7], can be applied.

1. Chiral ligand attachment

First, by controlling the amount of chiral ligand on the surface, the effect of ligand on CD signal generation was tested (Supplementary Figure 3a). Three different type of amino acid and peptide ligands, L-Cys, D-Cys, L-glutathione (GSH), were further attached by incubating each ligand (10 mM) for 5 min, washing, and repeating incubating and washing process for three times. This further attachment of the ligand only brings about slight intensity change without alteration of the spectral feature. Despite of attachment of different chiral molecules, D-Cys and L-GSH, the spectral features of CD were rarely changed and only intensity decrease observed. Also, further addition of L-Cys molecule on the surface, did not bring about enhancement of the g-factor. The reduced g-factor intensity in the L-Cys and D-Cys cases might be originated from the instability of the nanoparticles due to the shortening of the ligand by ligand exchange. When the L-Cys molecule detached by using $NaBH_4$ (10 mM $NaBH_4$ with 5 min incubation, washing, repeating 3 times), no significant g-factor change was observed. These marginal effect on the g-factor with the addition and detachment of chiral ligands indicate that the chiral ligand is not the main contributing factor for CD generation.

2. Embedment of chiral ligand

To prepare nanostructures in which chiral molecules are embedded, a pre-adsorption process is necessary. By adsorbing chiral molecules on the plasmonic nanoparticle and sequentially growing this pre-treated nanoparticle, the chiral molecules can be located or entrapped inside the plasmonic structure. To determine whether this mechanism plays a major role in chiral signal generation, we pre-adsorbed Cys molecule and grow this pre-treated seed in growth solution. First, we added Cys molecule (the same concentration with original synthesis process) into cuboctahedron particle and aged for 1 hour. Second, the pre-treated particle was grown in the growth solution without containing Cys molecule. At the stage of second growth, two type of pre-treated particle were prepared, one with the centrifuged to get rid of remaining Cys and one without centrifuged. Two different concentration of Cys were tested, but in all cases, only small g-factors and random shape of nanoparticle were observed (Supplementary Figure 3b and c). The deep dents observed in the nanoparticles are probably due to the adsorption of Cys. This difference depending on the preparation method suggests that embedding of molecules may not be the cause of CD signal generation.

3. Reduced g-factor at 0.3 mM Cys case

In the case of nanoparticle grown under the 0.3 mM Cys concentration (discussed in later part, Fig. 4), which is expected to include more Cys molecules on the surface than 0.2 mM and 0.1 mM case, the resultant particle shows significantly decreased g-factor (Fig. 4). In spite of the increased organic molecules in the plasmonic nanostructure, the result of a reduced g-factor suggests that a well-defined chiral structure plays an important role in determining the g-factor.

4. Effect of the CTAB and AA concentration on g-factor

We studied the effect of CTAB and ascorbic acid (AA) concentration on g-factor while the Cys concentration remains the same (Fig. 5a). The CTAB and AA are achiral ligands and these factors affect the dimension of nanoparticle, e.g. CTAB changes the distance between chiral edges and AA modifies the height of chiral edge (Supplementary Figure 14a). In this experimental set, the g-factors were changed more than 10-fold by changing the amount of this achiral ligand without changing any amount of Cys. The SEM images of the particles obtained by varying the CTAB and AA show that the g-factor varies greatly as the distance or height between the chiral edges changes, even if the degree of bending does not change. This experimental evidence directly shows that formation of defined structure is critical for chiroptical response.

Supplementary Discussion 2

In the case of 432 helicoid I, the cubic shape of seed nanoparticle is transformed into {321} polyhedral shape within 20 mins of growth time. The black line in Fig. 8b shows the crystallographic evolution of 432 helicoid I from {100} surface to {321} surface. At this point of growth, the Cys molecules are injected and started to interact with {321} high-index surface. The shift of edge become apparent after the 40 min.

The 432 helicoid II also showed a crystal structure which is close to the {321} index at the beginning of growth (10 min). However, since GSH was present and attached from the beginning, less growth was observed along the <100> direction and distorted edges were observable after 20 minutes of growth.

For the 432 helicoid III, the growth direction is different from the 432 helicoid I and II as it starts from octahedron seed composed of {111} planes. As the {111} plane is gradually added, the outline of the RD appears in 6 min of growth, and with further addition of {111} plane, distorted edges based on the $(22\bar{1})$ polyhedral shape appears.

In the case of 432 helicoid IV, since the growth was slowed by the doubled seed amount, the particle size was relatively small after 6 minutes of growth. The characteristic outline of the RD shows that the (100) face of the seed has been changed to the (110) facet. Cys, which is present at the beginning of growth, interacts with the crystallographic surface that appears in the transition between the (100) and (110) planes. Therefore, these differences create a distorted edge based on the RD outline.

Supplementary Discussion 3

Model construction

To build a structural model of 432 helicoid IV nanoparticles, we carefully analyzed the particles in all different directions based on SEM images. In the case of chiral nanoparticles synthesized under 0.1 mM cysteine, we observed characteristic outlines corresponding to a rhombic dodecahedron (RD) with slight distortion. Depending on the viewing direction of the RD, three different outlines, an elongated hexagon for the (110) axis, a hexagon for the (111) axis, and a square for the (100) axis, are observed (Fig. 2). The axis of the chiral nanoparticle was confirmed by TEM analysis. Based on the RD structure, we carefully designed chiral models by changing the edges of the nanoparticles. Three simplified models that describe the chiral twist of the edge in different ways were simulated and showed spectral features that corresponded well with experimental results (Supplementary Fig. 10).

Analysis of structural factors for optical activity

In this work, the diverse structural evolution of chiral nanoparticles was demonstrated. By varying the geometrical parameters in the simulation model, we studied the correlation between structural factors in nanoparticles with a chiroptical response. Models #2 and #3 in Supplementary Fig. 10 were selected in this structural-optical relationship study because of the ease of deformation in the chiral edge dimensions.

(a) Particle size (Supplementary Fig. 15b)

With increasing nanoparticle size, the extinction, CD, and g-factor linearly increased. As an increased particle size provides stronger dipolar modes and even high-order multipolar modes, this increase resulted in stronger extinction and chiro-optical responses.

(b) Core size (Supplementary Fig. 15c)

In this modification, only the size of the achiral core part was increased, while the width of the chiral structure remained the same. Although CD increased due to size increments, the g-factor decreased with increasing achiral core size. This result indicates that the proportion of the chiral edge in the entire nanostructure is important in achieving a high g-factor.

(c) Degree of bending of the edge (Supplementary Fig. 15d)

Increasing the degree of bending of the edge resulted in a significant change in the chiral response. While the extinction spectrum remained almost unchanged, the CD and g-factor were enhanced with the increase of the twist in the case of the #12 – #15 models. For example, a 16° increment of the bending angle led to a 3-fold enhancement of the g-factor. This trend explains the enhancement of the g-factor with increasing Cys concentration in the experimental results. However, in the case of the #16 model, a substantially decreased g-factor and CD spectra were observed. This result is probably due to strongly perturbed plasmonic modes and charge distribution near the two close (but opposite) chiral edges. Additionally, this might be the reason for the reduced CD intensity in the 0.3 mM Cys case.

(d) Protrusion of the chiral edge (Supplementary Fig. 15e)

As the chiral edge protruded, steep increases in CD and g-factor were observed. Increasing the height of the

protrusion not only increases the proportion of chiral structures in the particle but also increases the depths of the chiral gaps in the nanoparticles. The importance of the deep chiral gap in the CD signal was also pointed out in our previous paper.[8] This large enhancement of the g-factor with protrusion of the edge may originate from the strong dimeric coupling between two edges separated by the plasmonic gap. Increasing the height of the protrusion by a factor of 4 (changes from #17 to #20) resulted in a 10-fold increase in the g-factor. In a single parameter variation, an increased protrusion resulted in the greatest enhancement in the g-factor (Supplementary Fig. 15i).

(e) Width of the edge (Supplementary Fig. 15f and g)

To simulate the effect of the width, we tested two structural models with different modulations of the outer boundary. In both cases, widening the width of the edge by changing the curvature of the outer boundary resulted in enhancement of the g-factor. However, excessive expansion of the outer boundary led to a decrease in the g-factor, which might have originated from coupling of close edges, similar to the #16 model case.

(f) Combined modulation of the width and protrusion of the chiral edge (Supplementary Fig. 15h)

As shown in the experimental results, the combination of structural modulation and the degree of bending and protrusion brought about a synergistic effect on the g-factor increment. In this simulation study, we simultaneously increased the width of the chiral edge of the model and the value of the protrusion. Changing the width of the chiral edge under increased protrusion of the edge led to a significant enhancement of the g-factor. Interestingly, a new optical response with the highest g-factor was simulated in the case of model #32, where large modulations of two factors were applied. Based on the importance of the interplay of structural factors in strong g-factor, a combinatorial effect of the key structural factors on the chirality and experimental demonstration of the new structures are now under investigation.

REFERENCES

1 Urban, M. J. *et al.* Chiral Plasmonic Nanostructures Enabled by Bottom-Up Approaches. *Annu. Rev. Phys. Chem.* **70**, 275-299 (2019).

2 Ma, W. *et al.* Chiral Inorganic Nanostructures. *Chem. Rev.* **117**, 8041-8093 (2017).

3 di Gregorio, M. C., Ben Moshe, A., Tirosh, E., Galantini, L. & Markovich, G. Chiroptical Study of Plasmon–Molecule Interaction: The Case of Interaction of Glutathione with Silver Nanocubes. *J. Phys. Chem. C* **119**, 17111-17116 (2015).

4 Maoz, B. M. *et al.* Plasmonic Chiroptical Response of Silver Nanoparticles Interacting with Chiral Supramolecular Assemblies. *J. Am. Chem. Soc.* **134**, 17807-17813 (2012).

5 Hao, C. *et al.* Unusual Circularly Polarized Photocatalytic Activity in Nanogapped Gold-Silver Chiroplasmonic Nanostructures. *Adv. Funct. Mater.* **25**, 5816-5822 (2015).

6 Yan, J. *et al.* Fabricating Chiroptical Starfruit-like Au Nanoparticles via Interface Modulation of Chiral Thiols. *Nanoscale* **9**, 11093-11102 (2017).

7 Zheng, G. *et al.* Tuning the Morphology and Chiroptical Properties of Discrete Gold Nanorods with Amino Acids. *Angew. Chem. Int. Ed.* **57**, 16452-16457 (2018).

8 Lee, H. E. *et al.* Amino-acid- and Peptide-directed Synthesis of Chiral Plasmonic Gold Nanoparticles. *Nature* **556**, 360-365 (2018).
